# Research on an Intelligent Identification and Classification Method of Complex Holes in Triangle Meshes for 3D Printing

**DOI:** 10.1155/2022/2249925

**Published:** 2022-05-31

**Authors:** Shanhui Zhang, Wei Wei, Wei Wu

**Affiliations:** ^1^School of Control Science and Engineering, Shandong University, Jinan, Shandong 250061, China; ^2^Shandong Shanda Hoteam Software Co., Ltd, Jinan, Shandong 250101, China

## Abstract

In triangular mesh models, the repair of complex hole poses a difficult problem, which always causes serious repair defects. Therefore, it is needed to develop an intelligent identification and classification method of complex holes to reduce repair difficulties. First, the topological structure of the complex hole is studied and all the holes are divided into single holes and continuous holes depending on whether there are intersection points. Second, to tackle the nesting and connecting of complex continuous holes, a decomposition method of multiply connected domains based on intersection points is proposed to partition or reconstruct complex continuous holes into single holes. Based on the different geometric structures, single holes are classified into five common hole types and a corresponding identification method of single holes is presented. Finally, an experiment is carried out to verify the repair quality and efficiency of the proposed method. Compared with Geomagic software, the proposed method can automatically identify and partition complex holes with fewer defects and similar efficiency. It can reduce the difficulties of repairing complex holes and enable the repair of complex holes based on existing methods. It is shown that the method can be applied to complex hole repair of 3D printing models without the participation of technicians.

## 1. Introduction

As a rapidly developing manufacturing technology, 3D printing technology (also known as additive manufacturing technology or rapid prototyping technology) is gradually entering various fields and playing an important role [1, 2]. Compared with traditional manufacturing technology, 3D printing technology can better meet the modern green concept and presents a favourable trend in the green manufacturing industry. In 3D printing technology, triangle meshes are characterized by simplicity, straightforwardness, and expressiveness [3]. Thus, it becomes a widely used form of a geometric model and gradually proves to be an important basis of 3D printing technology. However, in the construction and acquisition of mesh models, holes are often generated, affecting the appearance and quality of 3D printed models. Therefore, the identification and repair of the holes in the triangle mesh model become the key to improving the quality of 3D printing.

At present, the identification and repair of triangle mesh holes are mostly carried out manually, with low efficiency and high requirements of operators' skills. Automatic hole identification and repair algorithms often regard all holes as single holes. Most research focuses on the repair method of single holes and disregards the processing and partitioning of complex holes, single hole geometry, and the topological relationship between relevant holes. This results in unsatisfactory model repair and new defects, such as self-intersecting surfaces, highly refracted edges, and degenerate triangles.

**Figure 1:**
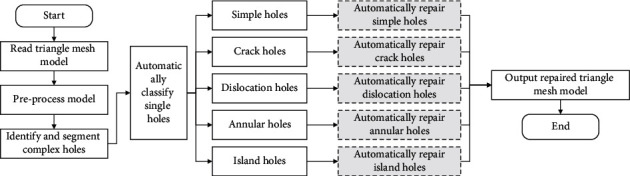
Technical route.

Therefore, to improve the quality of hole repair and to lower the reliance on specialized technicians, it is necessary to study the intelligent identification and partitioning method of complex holes in triangle meshes. The technical route of the paper is shown in Figure 1. First, the mesh model is checked and preprocessed. Second, the complex holes are identified and segmented and continuous holes are partitioned into single holes. Third, all the single holes are automatically classified into specific single holes, such as the simple hole, crack hole, and annular hole. Finally, the appropriate hole repair methods are chosen and the ideal triangle mesh model is output. The results show that the proposed method can accurately identify, partition, and repair complex holes with intersection points. The main contributions are summarized as follows:

1. A decomposition method of multiply connected domains based on intersection points is proposed to partition or reconstruct complex continuous holes into single holes. It can greatly improve the structure and size of the complex holes and can mostly avoid the intersection of various hole lines.
2. An identification method of single holes is researched to divide single holes into five common hole types, which can reduce the difficulties of repairing complex holes and enable the repair of complex holes based on existing repair methods of single holes.
3. The proposed repair process is completely automated, replacing the manual judgment and repair operation, reducing the dependence on professionals. At the same time, it can achieve high efficiency, similar to Geomagic.

## 2. Related Work

Hole identification and extraction is a critical preliminary process for hole repair. Given the considerable amount of data generated from the conversion from solid model to point cloud or grid, the data processing is time-consuming with low efficiency if holes are identified and extracted through repeated traversal. Therefore, the efficiency and accuracy of identification and extraction will directly affect the efficiency and quality of subsequent repair. The existing hole identification and extraction methods can be divided into two categories according to the data type: hole identification based on point cloud data and that based on grid [4, 5].

There are many methods for hole identification based on point cloud data [6–9]. Milroy et al. [10] identified the boundary of holes by calculating the extreme value of curvature. This method can generate good results in areas with small curvature changes and relatively smooth surfaces, but less so when the model curvature changed dramatically. Orriols and Binefa [11] adopted the least squares method to identify and query holes, which was also not suitable for drastic curvature changes. Bendels et al. [12] used the minimum spanning tree to detect feature points of the point cloud boundary, but the complete extraction of the boundary still needed to be further improved. Liu [13] calculated the barycentre of sampling points and that of sampling points in the neighborhood to efficiently extract the hole boundary according to the distance ratio of various points. Alrashdan et al. [14] used the neural network method to automatically extract the boundary points, but the boundary points with small change of normal vector were easily to be missed, so that the extracted boundary might not be complete, and it was also time-consuming.

The grid-based hole identification method is also a focal point of research. In computer graphics, grid is a very basic representation method, and it can be obtained by point cloud data reconstruction or 3D model transformation. Triangular mesh is the most widely used form because of its simplicity, straightforwardness, and expressiveness. Zhan and Zhang [15] proposed the area expansion method to identify hole boundary and to form the Delaunay triangulation of point cloud data. Chen et al. [16] projected the object model onto a two-dimensional plane and used the interior angles of the projected polygon in the plane and related theories to automatically identify the holes. Li et al. [17] proposed a hole identification method based on the winged-edge data structure, in which all the holes of the model can be obtained through one traversal, greatly improving the efficiency of hole identification. However, in practical 3D printing application, the point cloud data obtained by traversal may be unevenly distributed, which may easily lead to a situation where multiple holes share a boundary vertex [18]. Therefore, the above algorithms cannot accurately identify such complex holes, and it will cause repair defects, such as self-intersecting surfaces and structure change.

Based on the above hole identification methods, many scholars have conducted in-depth studies on the hole-filling of triangle mesh models, among which the typical repair methods were as follows: the feature plane is calculated based on the point distribution of holes and the points of holes were projected onto the feature plane for partitioning. Then, implicit function [19], radial basis function [20, 21], and the wave-front method [22] were used to map the points on the feature plane to the 3D model. The surface of the filled hole obtained by this method was often well shaped, but it was only applicable under extremely limited conditions. The method worked ideally for simple holes, but as for complex holes, mapping failure or errors may occur, leading to inferior hole filling. Therefore, a large number of scholars have proposed the classification and filling methods for complex holes.

Jun [23] proposed an algorithm of subdividing complex holes into simple holes before repairing them, providing a solution for the subdivision and repairing of holes with self-intersecting boundaries in the projection process. However, it was not conducive to dealing with other types of complex holes, especially with the challenge of self-intersecting boundaries in the nonprojection process. Inspired by this method, Li et al. [17] presented and created an algorithm based on edge expansion, which split the holes into flatter ones considering the spatial shape of holes. The method cannot deal with self-intersecting boundaries, and the complex holes were in fact just a simple hole with complex curvature changes. Lai et al. [24] mulled the processing of island holes and proposed a hole filling algorithm based on B-spline surfaces, fitting the vertices near holes to B-spline surfaces. This method focused on the topological accuracy and smoothness of the connection between new meshes and existing ones. Feng et al. [25] proposed a fast filling method for triangle meshes based on the hole size. The holes were classified into small holes, medium holes, and large holes according to their size, and different filling algorithms were used for different types of holes. However, the classification method only factored in the size of holes while ignoring the complex topological form of holes, thus leading to subpar filling results for complex holes. Wen et al. [26] proposed a method for automatic identification and repair of real defect holes, but it was only applicable in simply connected domains and ignored the topological form of the complex missing area. Therefore, the above studies lack the identification and segmentation of complex multiconnected domain holes, especially of the holes with self-intersecting boundaries.

To sum up, the repair methods for a specific type of holes, especially for simple holes, are relatively sophisticated, having delivered good repair results in previous research. However, the identification and partitioning of complex holes and the corresponding repair methods vary with each case. Adopting a uniform repair method will lead to self-intersecting surfaces in hole repair and unsatisfactory repair outcomes. At the same time, the human-computer interaction is often adopted in hole repair with ultralow identification and repair efficiency. In this case, the repair outcomes depend on the precision of the repair algorithm and the operator's skills. In order to solve this problem, based on the topological form of holes, ways must be found to intelligently identify and partition complex holes with multiconnected domains and self-intersecting boundaries. Thus, a method of automatically detecting, identifying, and partitioning of holes in triangle mesh models should be proposed, aiming at the nested and crossed characteristics of complex continuous holes. It can make up for the deficiency of various simple hole repair methods in complex holes.

## 3. Identification and Partitioning of Complex Holes

### 3.1. Features of Single Holes and Continuous Holes

Through compiling and analyzing the data of hole repair failure cases in the triangle mesh model, it is found that the failure often occurs when multiple holes are adjacent or nested to each other. It leads to mapping failure and thus unsatisfactory repair outcomes. In order to solve this problem, it is necessary to analyze the characteristics of such holes and differentiate them from single holes. Through analyzing many cases, it is found that the main difference is whether there are intersection points connecting multiple hole lines. On this basis, all the holes are classified into two types.

**Figure 2:**
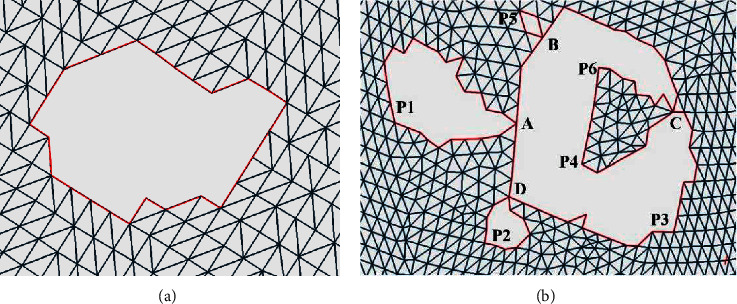
Examples of (a) single hole and (b) continuous hole.

1. Single holes: the points on the hole line can be connected in turn to form a closed loop with a unique solution. This is shown in Figure 2(a).
2. Continuous holes: a continuous hole consists of multiple single holes, and there are one or more intersection points between the single holes. It offers multiple ways of representing each individual closed loop. As shown in Figure 2(b), four points A, B, C, and D of the continuous hole are the intersection points of multiple hole lines. When the counterclockwise direction is defined positive, the continuous hole boundary can be identified as A-P1-A-B-P5-B-C-P6-P4-C-P3-D-P2-D-A.

### 3.2. Identification of Single Holes and Continuous Holes

Because of the large difference between repairing single holes and continuous holes in triangle meshes in terms of difficulties and methods, it is an essential step to classify and identify all holes and determine whether they are single holes or continuous holes before hole repair. Before identification, it is necessary to preprocess the input triangle mesh model and quickly establish the topological structure of the model's facets, edges, and vertices. According to the relationship between facets and edges of the triangle mesh model, all simply connected domains of the triangle mesh model are obtained, and each simply connected domain is regarded as a part. Free edges are found for each part, and all free edges are connected from end to end according to the connection between edges and points to obtain hole lines, so the basic information of all holes is obtained, including hole lines, hole directions, and the relationship between holes and parts. Among them, a free edge refers to an edge connected with only one facet. A hole line refers to the closed line formed by connecting the ends of free edges in each set, maintaining the topological relationship of the original triangle mesh model.

**Figure 3:**
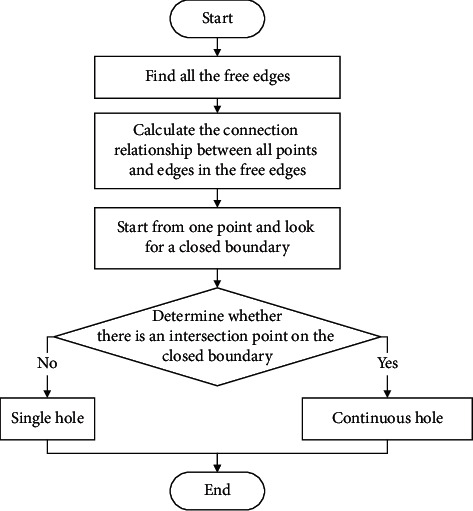
Identification process of single holes and continuous holes.

On this basis, an identification process of single holes and continuous holes is put forward, as shown in Figure 3. First, all the free edges of the hole lines are found by obtaining the basic information of the hole. The sets of free edges are discovered. Second, according to the topological structure relationship between each edge and each vertex, the connection between all points and edges in the free edges is calculated. Third, starting from any point, the closed boundaries are found according to the relation of connection. Then, it is judged whether there is an intersection point on the closed hole lines. If not, a single hole is output; if so, it is a continuous hole, and the corresponding boundary information is output to facilitate the subsequent partitioning of continuous holes.

### 3.3. Segmentation Method of Continuous Holes

**Figure 4:**
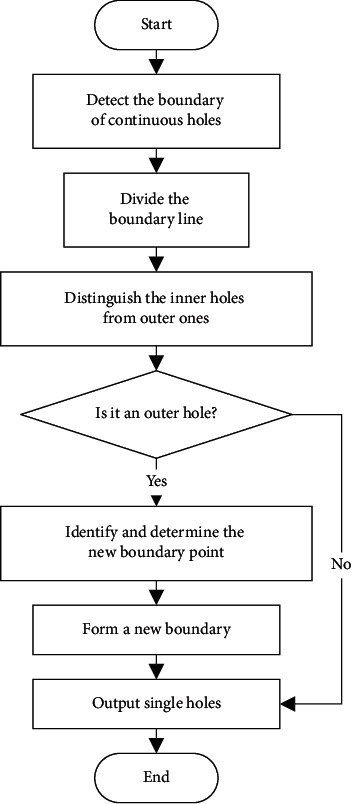
Segmentation process of continuous holes.

In order to facilitate the repair of triangle mesh models, the complex continuous holes must be divided into single holes to improve the accuracy and repair quality of each single hole repair algorithm. The nesting and connection of the hole lines in continuous holes are likely to cause errors in the partitioning of multiple holes. To solve this problem, a decomposition method of multiply connected domains based on intersection points is proposed to segment and reconstruct the hole lines of continuous holes. The specific steps are shown in Figure 4.

**Figure 5:**
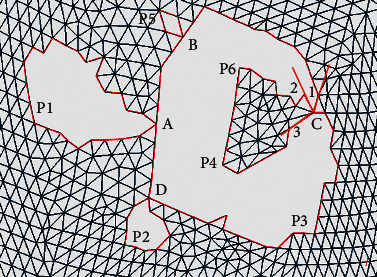
Determination of the subsequent boundary point of an outer hole line.

**Figure 6:**
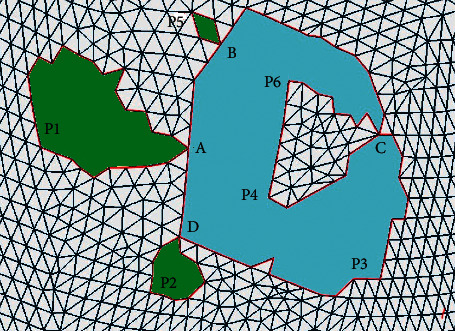
Single hole identification and output.

  Step 1: detect the boundary of continuous holes.
  Based on the information obtained during identification, the boundary of the continuous hole is detected, and the corresponding hole line is formed in the counterclockwise direction. For example, the continuous hole boundary in Figure 2(b) is A-P1-A-B-P5-B-C-P6-P4-C-P3-D-P2-D-A, and four intersection points A, B, C, and D are identified.
  Step 2: divide the boundary line.
  According to the intersection points of continuous holes, the inner area of continuous holes is divided into several separate holes. For example, the boundary of continuous holes in Figure 2(b) can be divided into five separate holes, namely, A-P1-A, B–P5–B, C–P6–P4–C, D-P2-D, and A-B-C-P3-D-A.
  Step 3: distinguish the inner holes from outer ones.
  The points on the hole line are projected onto the plane, and the positional relations of each hole are calculated according to the relative inner and outer relations between the points and the polygon. If the points of a hole L1 are all outside of another hole L2, it is assumed that L1 is outside of L2. If a hole line is not inside any of the holes, it is an outer hole, and if a hole line is inside one of the holes, it is an inner hole.
  Step 4: judge whether it is an outer hole.
  If the outer hole does not contain the inner hole, jump to Step 7; otherwise, continue with Step 5. It can be concluded that the outer holes in the continuous holes in Figure 2(b) are A-P1-A, B–P5–B, D-P2-D, and A-B-C-P3-D-A and the inner hole is C–P6–P4–C, which is included by the outer holes A-B-C-P3-D-A.
  Step 5: identify and determine the new boundary point.
  For an outer hole that contains an inner hole, the intersection point in its hole line must be found, and there are three important edges connected to the intersection point, namely, the first edge connecting to the previous index point of the current outer hole line and the second and third edge connecting to two neighbouring index points in the inner hole line. Calculate the included angle between the first edge and the second and third edge. Select the neighbouring index point with the smallest included angle as the new boundary point. For example, Figure 5 shows that C1, C2, and C3 are the three important edges, and the included angle ∠1C2 is less than angle ∠1C3. Thus, the next boundary point is calculated to be in the direction of ray C2, that is, in the direction of P6, rather than P4.
  Step 6: form a new boundary.
  Starting from the new boundary point, all the points on the inner hole are inserted into the boundary of the outer hole in accordance with the connection order on the boundary, and the outer and inner edges of the hole are combined. In other words, in the example, the inner hole C–P6–P4–C is inserted into the outer hole A-B-C-P3-D-A and the new boundary line is A-B-C-P6-P4-C-P3-D-A.
  Step 7: output single holes.
  All the holes that meet the conditions are output as a single hole, with the boundary lines given. According to this method, the example outputs four single holes with the boundary lines A-P1-A, B–P5–B, D-P2-D, and A-B-C-P6-P4-C-P3-D-A. The filled area as shown in Figure 6 is four single holes surrounded by four boundary lines.
  Step 8: end.

**Figure 7:**
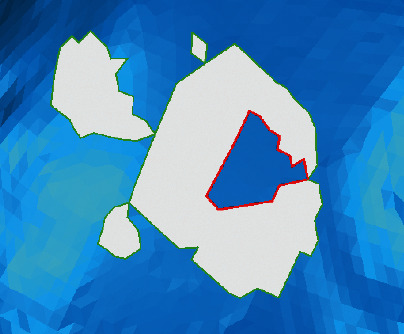
Hole identification result of Geomagic.

When the hole in this case is processed by traditional hole identification methods and software such as Geomagic, C–P6–P4–C can be easily identified and be output as a single hole. If filled based on this, the model obtained will not be desirable and a large number of self-intersecting surfaces will be created. As shown in Figure 7, both the green and red hole lines are identified as single holes, but the inner red hole line will intersect with the outer hole line during repair, obviously inconsistent with the actual hole.

## 4. Types of Single Holes and the Intelligent Identification Method

### 4.1. Types of Single Holes

**Figure 8:**
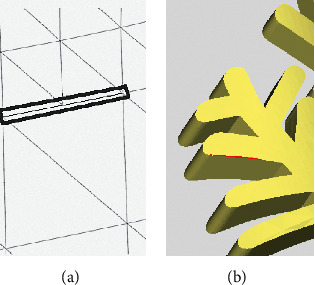
Crack hole. (a) Triangle mesh example. (b) Model example.

**Figure 9:**
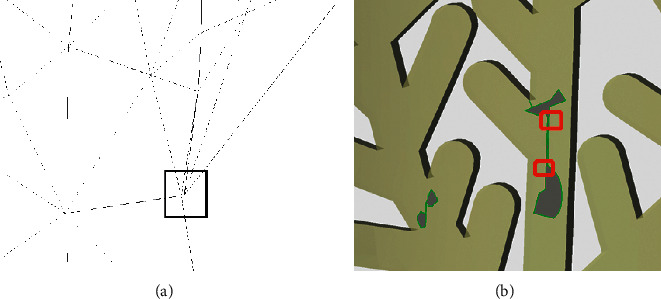
Dislocation hole. (a) Triangle mesh example. (b) Model example.

**Figure 10:**
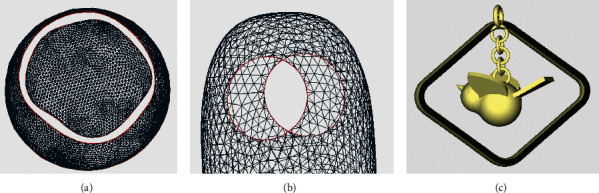
Annular hole. (a) Triangle mesh example—coplanar annular hole. (b) Triangle mesh example—parallel annular hole. (c) Model example.

**Figure 11:**
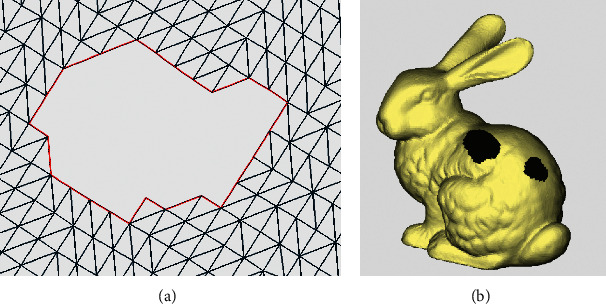
Simple hole. (a) Triangle mesh example. (b) Model example.

**Figure 12:**
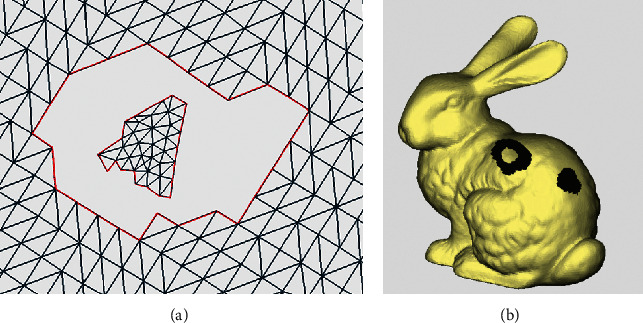
Island hole. (a) Triangle mesh example. (b) Model example.

After the complex continuous holes are divided into single holes, the geometry of each single hole still varies, posing challenges to the hole repair process. Therefore, by analyzing causes of self-intersecting surfaces based on the geometry of the hole and the current sophisticated hole repair methods, the single hole is further classified into five types: crack hole, dislocation hole, annular hole, simple hole, and island hole. Their respective characteristics are as follows:

1. Crack hole: if the included angle and the cumulative included angle of any two sides forming the hole boundary are less than the set angle threshold, such a single hole is defined as a crack hole. Its shape resembles a straight line. The general rule of thumb is that the angle threshold is set between 5° and 10°, which is used to determine whether the hole resembles a straight line. The cumulative angle refers to the sum of angles between all boundary edges starting from the first edge. Crack holes, which occur in modelling or format conversion, are linear bad edges that are not sewn together at the surface joints. There are no triangles missing, but the adjacent triangles are not connected, as shown in Figure 8. Therefore, the crack holes are more suitable for repairing by stitching the boundary points and then optimizing the subdivision.
2. Dislocation hole: if the distance between the points on the hole edge and on the nearest edge is very short (less than the set distance threshold), such holes are called dislocation holes, as shown in Figure 9(a). The distance threshold is also an empirical value and is generally set to about 0.1–0.5 mm. If it is less than this value, the hole is fixed by kneading the triangles together, generally without affecting the shape of the model; if it is above the set threshold, the hole is considered as a simple hole and can be repaired by adding triangle surfaces, which will not cause too many narrow and long triangles. As shown in Figure 9(b), both the red parts are dislocation holes. After judgment, the holes can be partitioned into simple holes and crack holes by merging the surfaces of dislocation holes.
3. Annular hole: it refers to a hole composed of two boundary lines, nearly coplanar or parallel, with similar shapes and without intersection points, as indicated in Figure 10. There is an approximate coplanar or parallel relationship between the positions of the two annular closed curves, which is generally seen in the cross section or the profile of the model. The repair method of firstly stitching two hole boundaries and then optimizing the triangulation and fitting the surface is suitable.
4. Simple hole: it refers to a single hole with no triangle surface data inside and only one closed boundary, as shown in Figure 11. It is the simplest type of single holes, and it is the type of hole that most repair methods are applicable to.
5. Island hole: it refers to the existence of an independent grid area composed of triangle surfaces inside a hole, as shown in Figure 12. This individual grid area is called an island, and the number of islands can be one or more. According to the size of the islands, different repair methods can be selected, such as the multidirectional advancing method [27] and variational implicit surfaces [4].

### 4.2. Intelligent Identification Method of Single Holes

**Figure 13:**
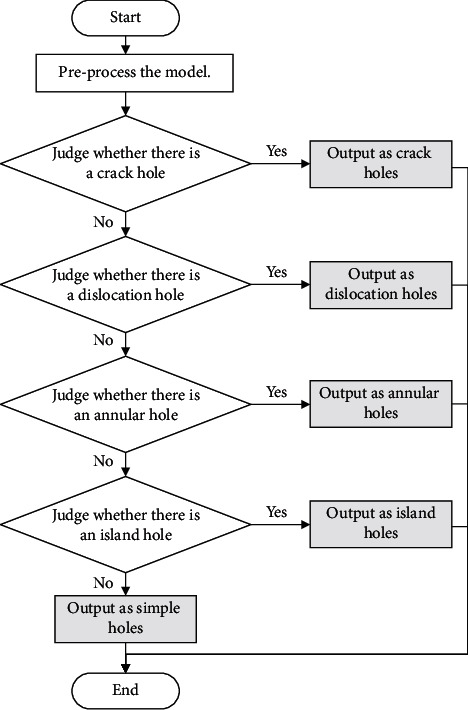
Identification process of single holes.

After the identification and the segmentation of complex holes into single holes, the shapes of single holes are still different. To prevent various repair defects, different repair methods should be adopted according to hole types. Therefore, an intelligent identification method of single holes is researched to avoid human errors, which can judge the types of five holes in turn and realize the automatic and accurate identification of single holes. The specific judgment process is shown in Figure 13. It is described as follows.

  Step 1: preprocess the model.
  Traverse all the parts of the triangular model and output the basic information of every single hole, for instance, vertexes, free edges, and boundaries. This step provides a data basis for other steps.
  Step 2: judge whether there is a crack hole.
  Calculate the included angle and the cumulative included angle of any two edges that constitute the single hole. If the value is less than the set threshold value, output the hole as a crack hole; if not, proceed to Step 3.
  Step 3: judge whether there is a dislocation hole.
  Calculate the distance between points on the single hole and on their nearest edges. If it is less than the set distance, the current point is the dislocation point and output the hole as a dislocation hole; if not, continue with Step 4.
  Step 4: judge whether there is an annular hole.
  Determine whether there are two boundaries that are the closest and most similar to each other and whether the gravity center of one boundary is within another boundary on the fitting plane. If so, output the hole as an annular hole; if not, proceed to Step 5.
  Step 5: judge whether there is an island hole.
  Determine whether there are one or more boundaries that are inside another boundary. If so, output it as an island hole; if not, output it as a simple hole.
  Step 6: end.

## 5. Experimental Results and Discussion

As few studies have been conducted on the classification and identification method of the complex holes with intersection points in triangle mesh models, it is difficult to find a targeted algorithm for comparison. For this reason, the well-known commercial software Geomagic is selected to determine the identification and repair effect of the proposed method. All the developed algorithms are implemented in C++ by using Visual Studio 2013 and tested on a PC equipped with an Intel^®^Core i7-4790 processor and 8 GB of RAM on Windows 10. In the process of comparison, various proven methods in repairing different single holes can be chosen. Crack holes and dislocation holes are repaired through the suture or kneading of the corresponding points. Annular holes, simple holes, and island holes are repaired with the variational implicit surface algorithm [28–30].

**Figure 14:**
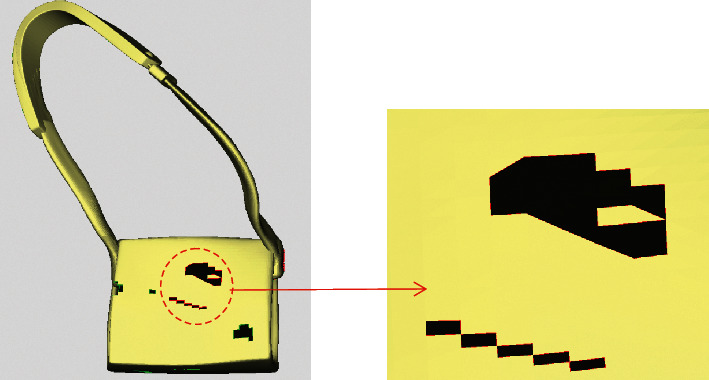
Backpack example model.

Taking the backpack model in Figure 14 as an example, the model has a complex hole, including multiple single holes and complex continuous holes. All single holes and continuous holes can be identified by the proposed hole identification method. The red lines represent continuous holes, and the green lines represent single holes. Two sets of continuous holes are identified, that is, the area surrounded by dash-dotted lines. By means of the continuous hole segmentation method, the upper continuous hole can be merged into a complete single hole and the lower continuous hole can be divided into five simple holes. Therefore, it has been verified that this method can be applied to identify and to partition complex holes. It can provide effective preprocessing that facilitates subsequent hole repair.

**Figure 15:**
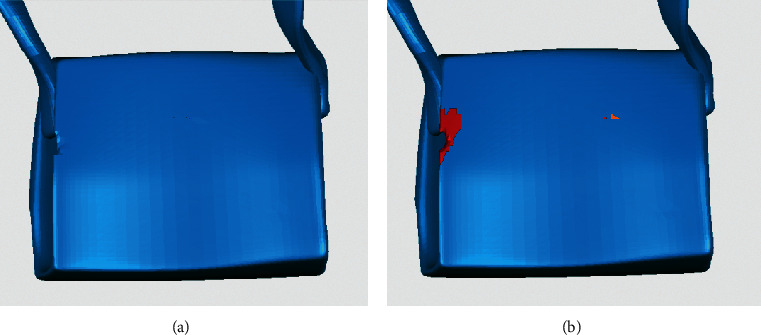
Comparison of repair results—continuous holes of a backpack model. (a) Repaired with the proposed method. (b) Repaired with Geomagic.

**Figure 16:**
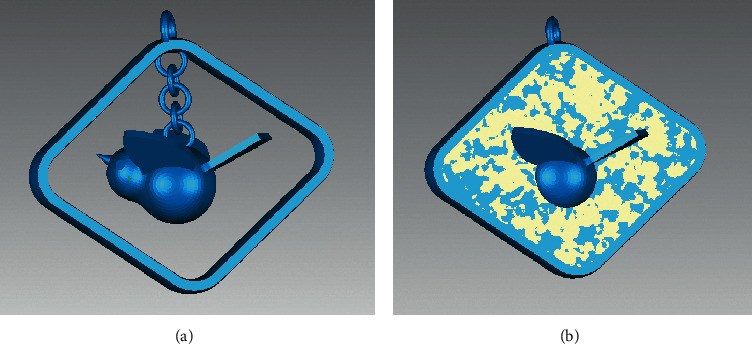
Comparison of repair results—annular holes of a bird pendant model. (a) Repaired with the proposed method. (b) Repaired with Geomagic.

The repair result of the algorithm proposed in this paper is compared with Geomagic. In terms of the identification and repair of continuous holes, the proposed method can accurately complete the complex hole segmentation and repair, but the commercial software generates multiple spikes and a large number of highly refracted edges, as shown in Figure 15. In terms of the identification and repair of annular holes, the model is corrected after being repaired with the proposed method and many self-intersecting surfaces are produced with Geomagic, which destroy the original structure of the model, as shown in Figure 16.

**Figure 17:**
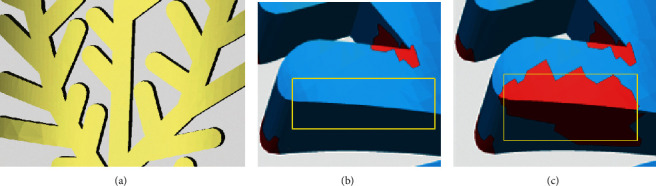
Comparison of repair results—crack holes and dislocation holes of a leaf model. (a) Repaired with the proposed method. (b) Enlarged view repaired with the proposed method. (c) Enlarged view repaired with Geomagic.

In terms of the identification and repair of crack holes and dislocation holes, as shown in Figure 17, the repaired crack holes and dislocation holes are located in the yellow box. The model structure is normal after being repaired by the proposed method, and the repaired region does not produce self-intersecting and highly refractive edges. After the repair of Geomagic, a number of self-intersecting surfaces, highly refractive edges, and surrounding degenerate triangles, indicated by the red area, are generated. After the degenerate triangles are deleted, it has been found that there are obvious degenerate surfaces in the repaired model, which is not conducive to the subsequent editing and 3D printing of the model.

**Figure 18:**
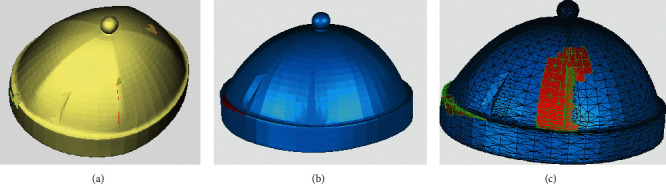
Comparison of identification and repair results—complex holes of a hat model. (a) Original model with holes. (b) Repaired with the proposed method. (c) Repaired with Geomagic.

**Figure 19:**
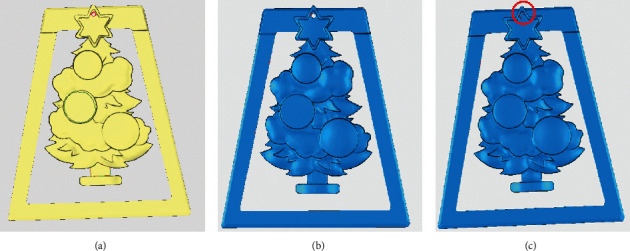
Comparison of identification and repair results—complex holes of an ornament model. (a) Original model with holes. (b) Repaired with the proposed method. (c) Repaired with Geomagic.

For the identification and repair of complex holes, the hat model and the ornament model are also chosen for validation, as shown in Figures 18 and 19. In the hat model, eight crack holes and three simple holes were identified and partitioned. In the ornament model, one annular hole, two simple holes, and one island hole were identified. After using the identification and repair methods in this paper, the models are all better treated. By contrast, there is an obvious error in the repair of annular holes with Geomagic for the ornament model. The error is circled by a red line, as shown in Figure 19.

To accurately check the repair quality, the above two repaired models were scanned by a grid doctor. In terms of the six types of repair defects, including nonmanifold edges, self-intersections, highly-creased edges, spikes, small components, and small holes, Geomagic is found to generate more defects such as spikes, highly creased edges, and self-intersections, while the model repaired by the proposed method has fewer defects and a higher repair quality. The repair time of the proposed method is equivalent to Geomagic; thus, the repair efficiency is acceptable. Specific comparison information is shown in Table 1.

Through the above examples, it can be found that the complex continuous holes containing intersection points can be segmented and all kinds of holes can be accurately identified with the proposed method, as shown in Table 2. Subsequently, the existing mature methods can be used to repair corresponding holes and the repaired models have fewer repair defects and a higher repair quality. However, some of these holes cannot be well segmented and identified with Geomagic without the participation of professionals. The proposed repair process is completely automated, replacing the manual judgment and repair operation, reducing the dependence on professionals.

## 6. Conclusion

In this work, an intelligent identification and classification method of complex holes is proposed. It can identify, partition, and reconstruct complex holes into single holes based on the intersection points connecting multiple hole lines and classify all the single holes into five types, such as crack holes, dislocation holes, annular holes, simple holes, and island holes. Thus, proper hole repair algorithms can be selected according to the type of the single holes. Compared with Geomagic software, the proposed method can mostly avoid the intersection of various hole lines and reduce the defects of traditional hole repair methods, but it can achieve similar efficiency. The proposed method provides a new idea for the automatic hole repair of triangle meshes without the participation of professionals and can be widely used in 3D printing model repair.

In future work, first, the identification method of boundary holes that do not have closed hole lines should be researched. Second, it is recommended to select and study more accurate repair algorithms for some single holes with large curvature variations to further improve the repair results.

## Figures and Tables

**Table 1:** Comparison of repair results after inspection.

Models	Methods	Nonmanifold edges	Self-intersections	Highly creased edges	Spikes	Small components	Small holes	Repair time (s)
Hat	Geomagic	0	100	64	24	0	0	0.093
	The proposed method	0	0	0	12	0	0	0.090

Ornament	Geomagic	0	188	113	0	0	0	0.082
	The proposed method	0	0	26	0	0	0	0.086

**Table 2:** Comparison of hole identification and repair results.

Models	Methods	Continuous holes	Simple holes	Crack holes	Dislocation holes	Annular holes	Island holes
Backpack	Geomagic	×	√	—	—	—	—
	The proposed method	√	√	—	—	—	—

Bird pendant	Geomagic	—	—	—	—	×	—
	The proposed method	—	—	—	—	√	—

Leaf	Geomagic	—	—	×	×	—	—
	The proposed method	—	—	√	√	—	—

Hat	Geomagic	—	√	×	—	—	—
	The proposed method	—	√	√	—	—	—

Ornament	Geomagic	—	√	—	—	×	√
	The proposed method	—	√	—	—	√	√

“×” represents that the model has such holes and the method is not workable; “√” represents that the model has such holes and the method is workable; “—” represents that the model does not have such holes.

## Data Availability

The data used to support the findings of this study are included within the article.
